# Supplementary figures and images for: Amylopectinosis of the fatal epilepsy Lafora disease resists autophagic glycogen catabolism
Source: EMBO Mol Med. 2024 Apr 2;16(5):1047–50. doi: 10.1038/s44321-024-00063-9 (PMC11099118; doi:10.1038/s44321-024-00063-9)

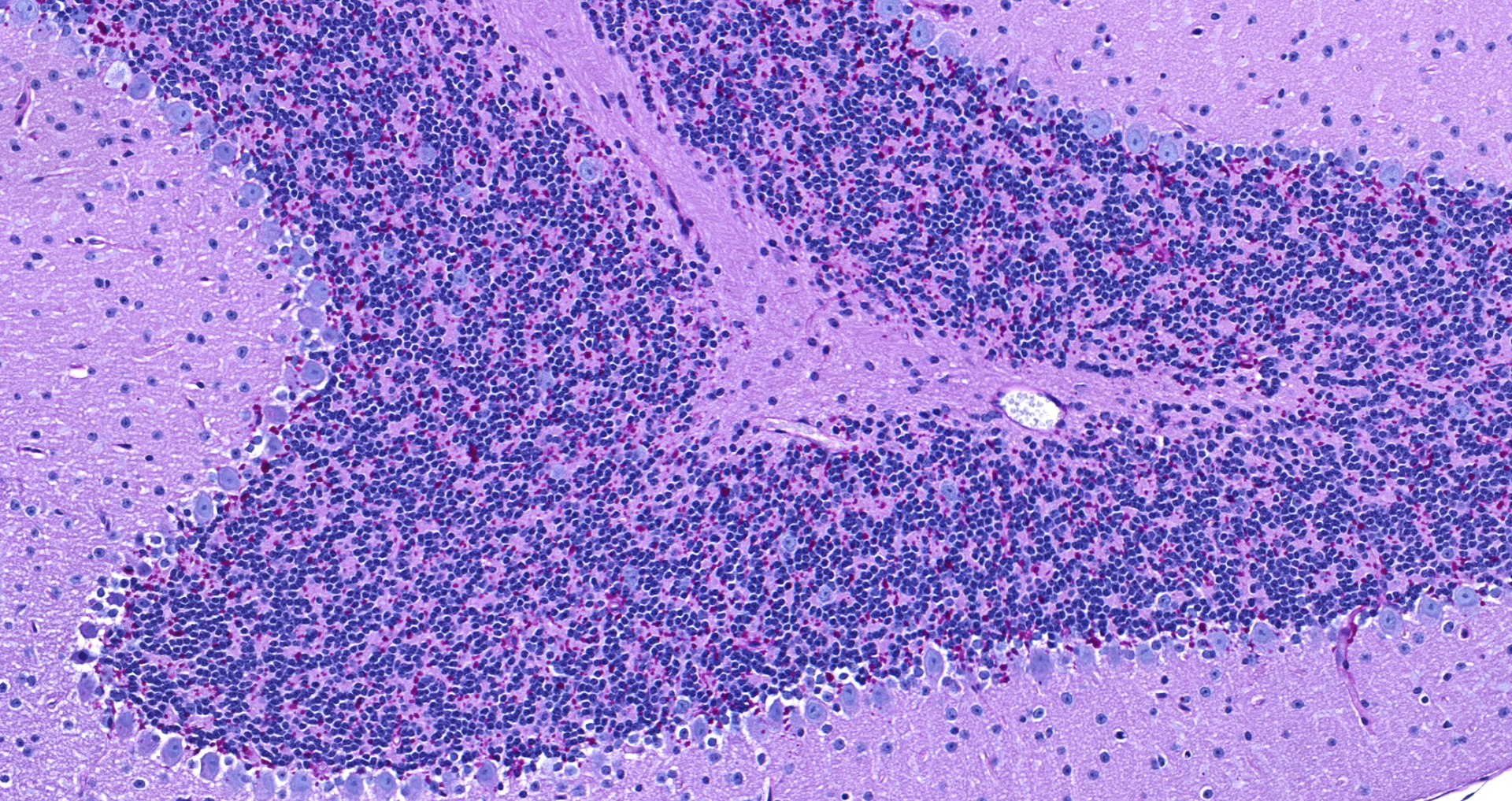

Supplement: Supplementary file 1 — Source data Fig. 1 [file 44321_2024_63_MOESM1_ESM.zip › Figure 1/Figure 1A Image data/Cerebellum - Control.jpg]

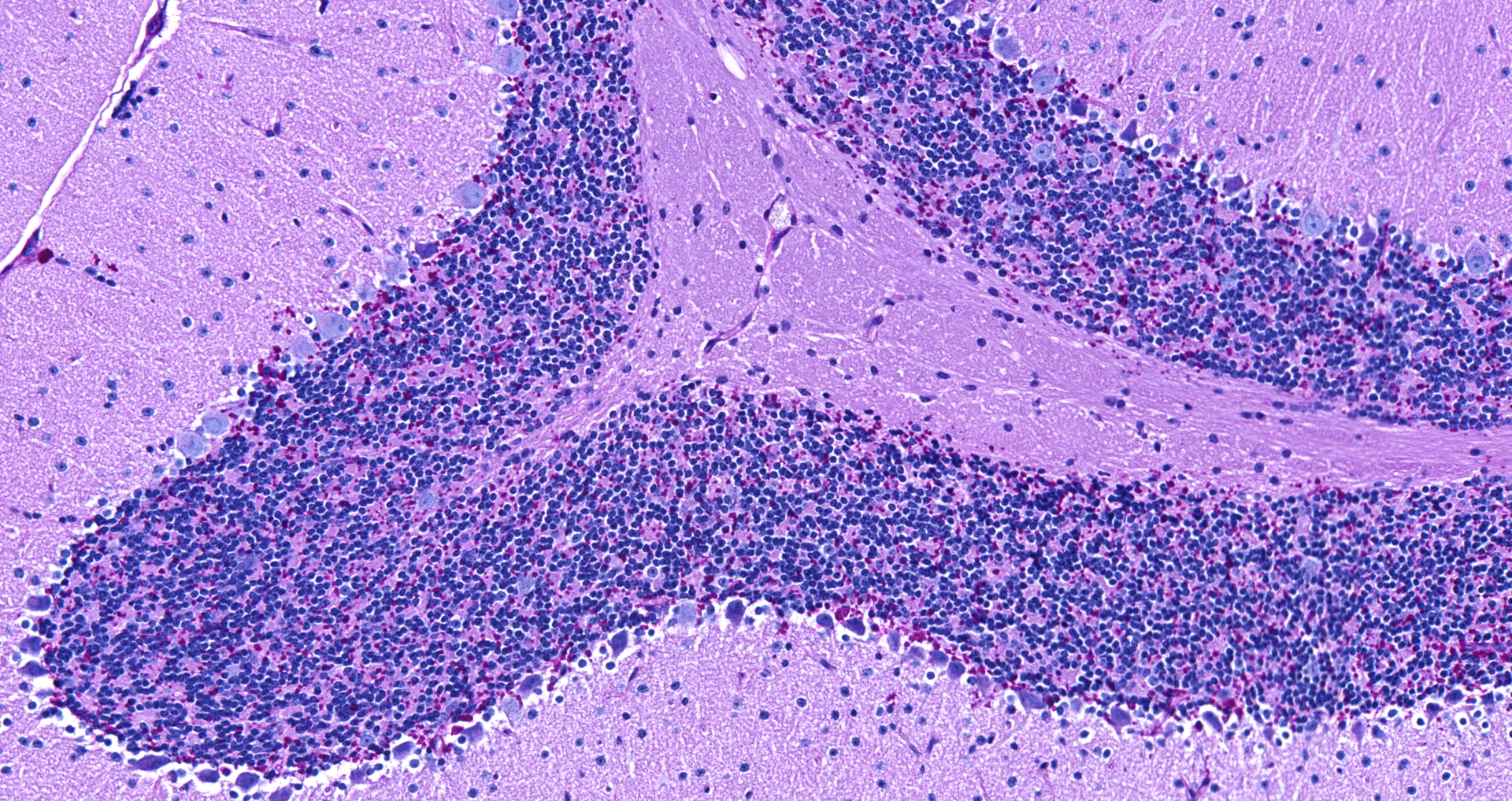

Supplement: Supplementary file 1 — Source data Fig. 1 [file 44321_2024_63_MOESM1_ESM.zip › Figure 1/Figure 1A Image data/Cerebellum - GHF201.jpg]

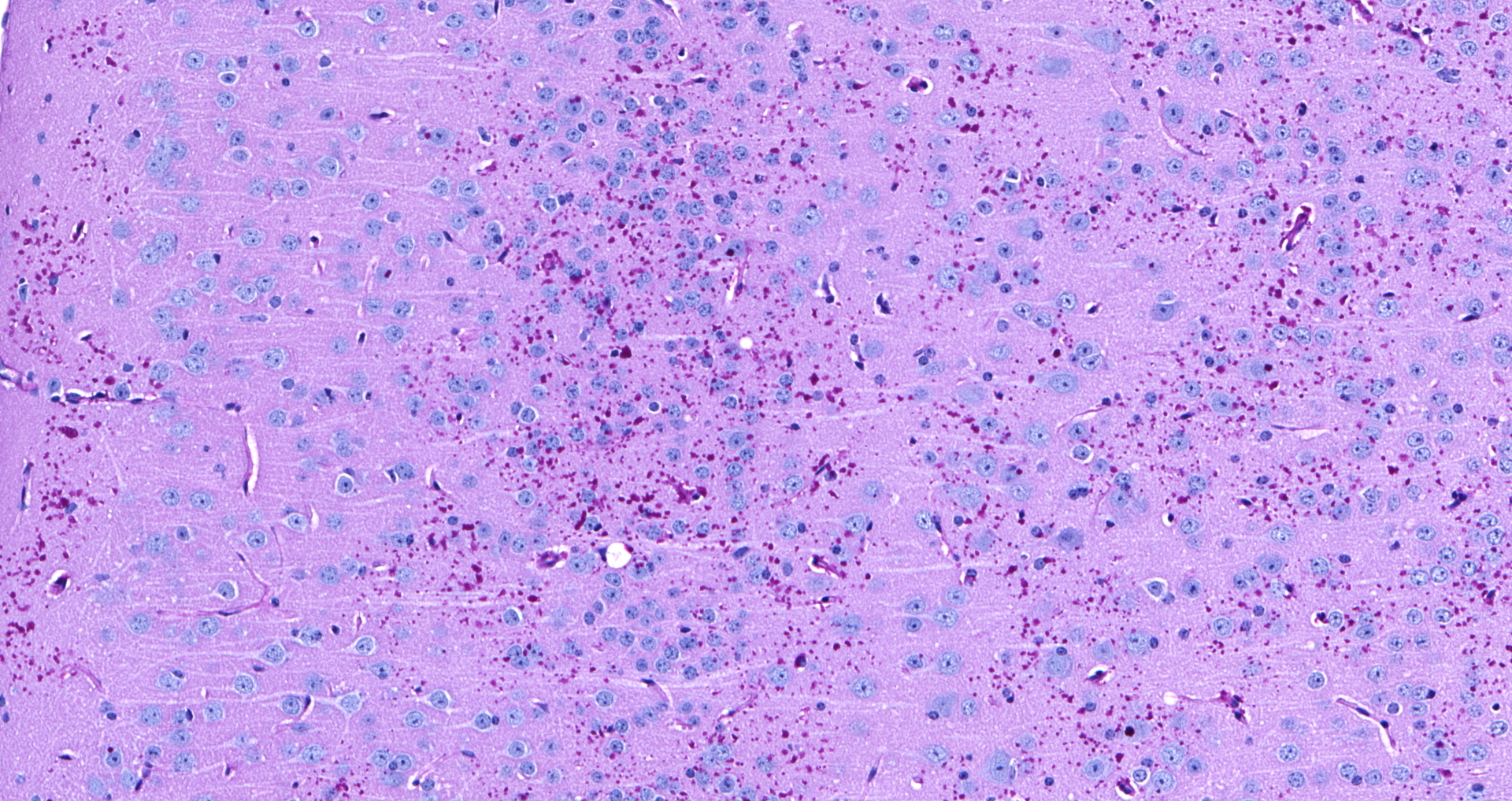

Supplement: Supplementary file 1 — Source data Fig. 1 [file 44321_2024_63_MOESM1_ESM.zip › Figure 1/Figure 1A Image data/Cortex - Control.jpg]

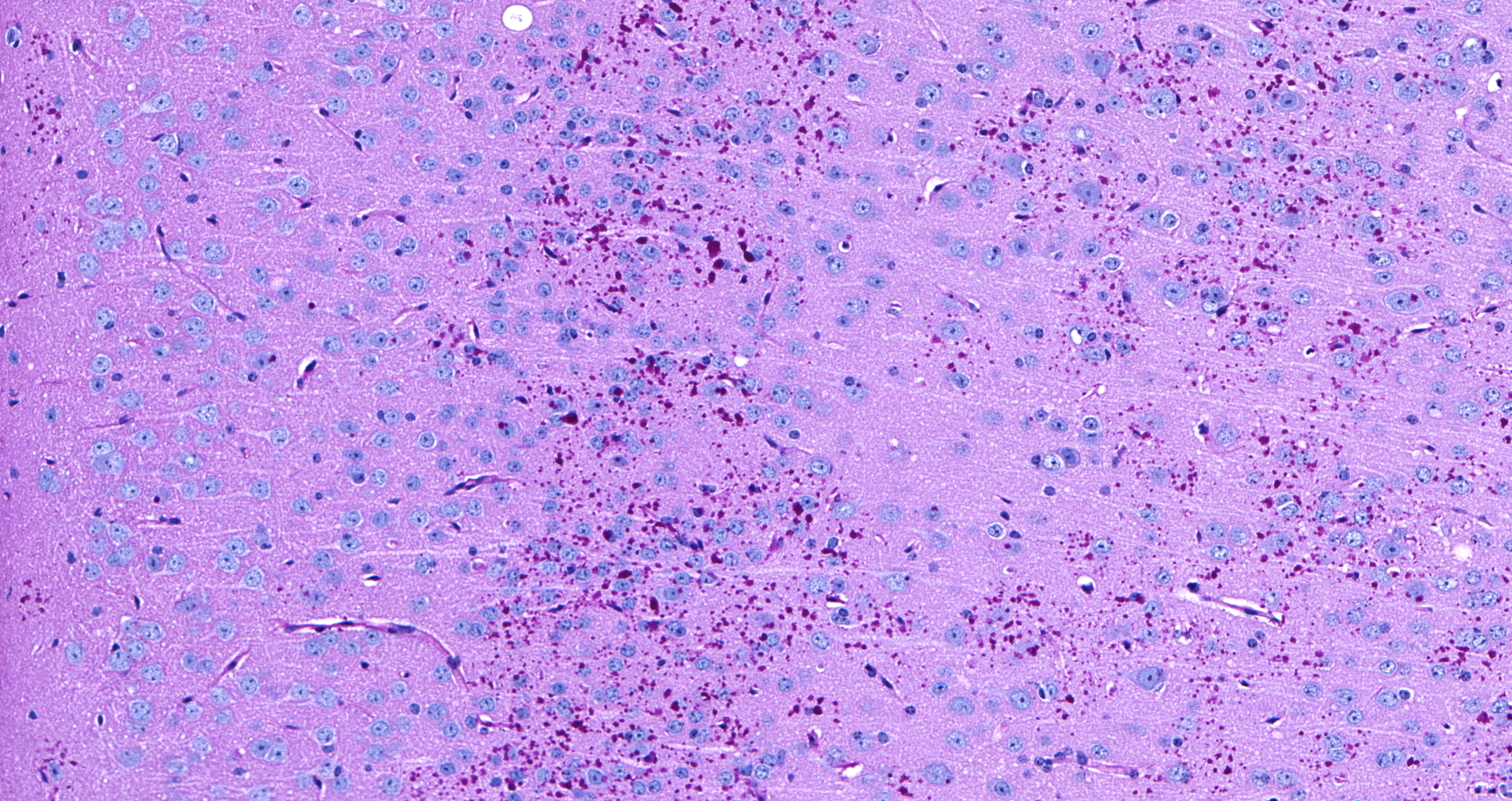

Supplement: Supplementary file 1 — Source data Fig. 1 [file 44321_2024_63_MOESM1_ESM.zip › Figure 1/Figure 1A Image data/Cortex - GHF201.jpg]

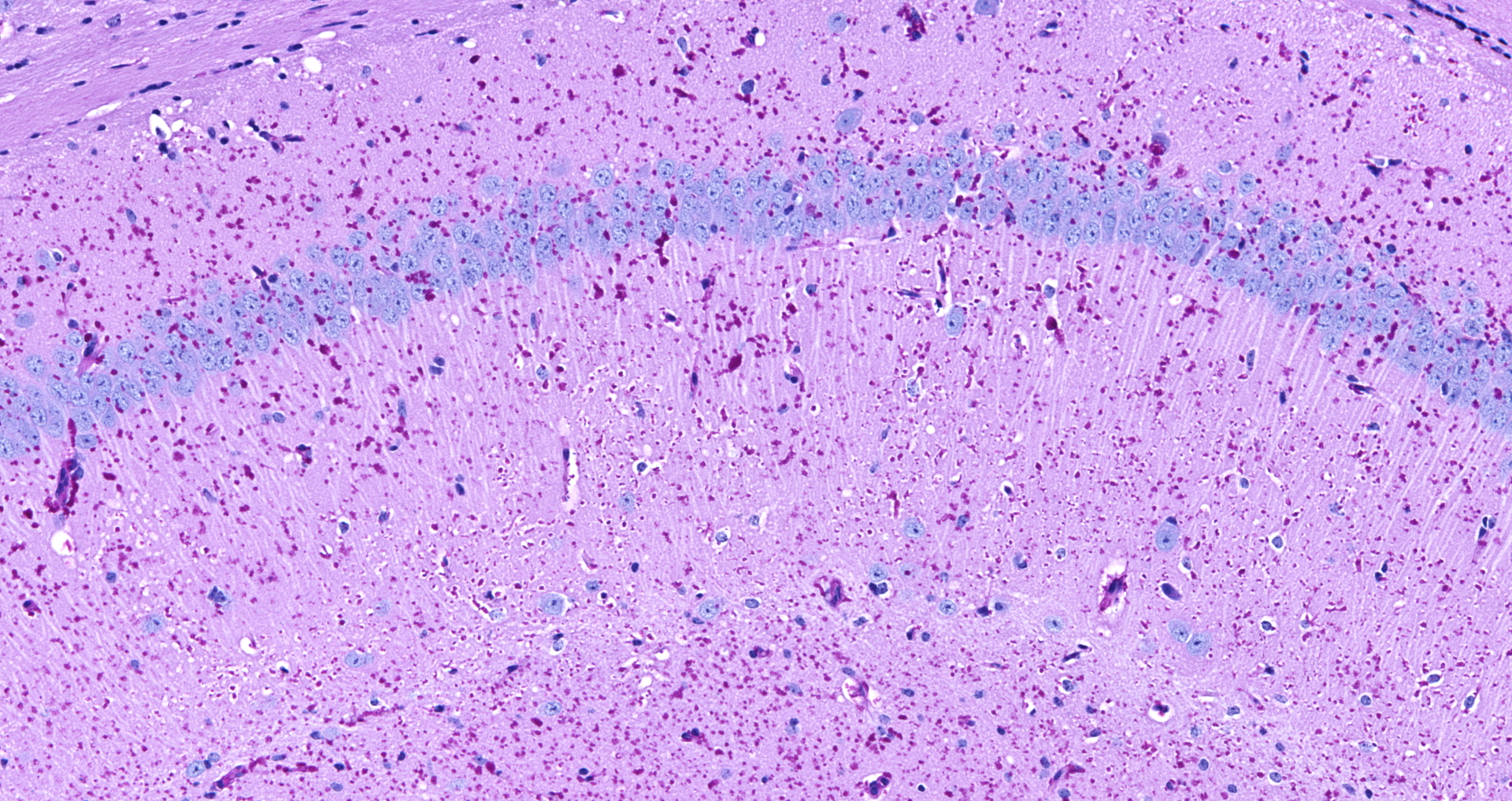

Supplement: Supplementary file 1 — Source data Fig. 1 [file 44321_2024_63_MOESM1_ESM.zip › Figure 1/Figure 1A Image data/Hippocampus CA1 - Control.jpg]

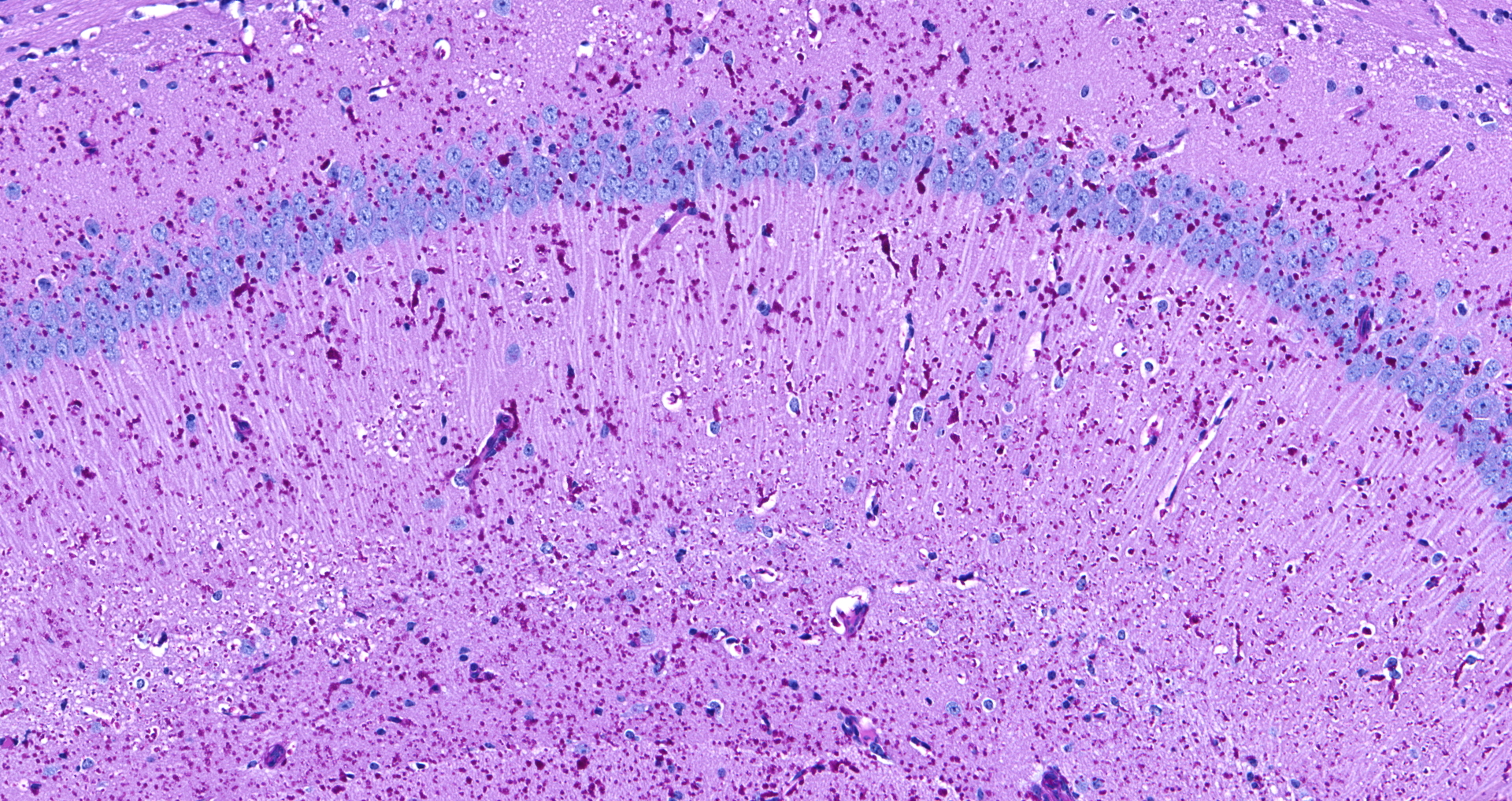

Supplement: Supplementary file 1 — Source data Fig. 1 [file 44321_2024_63_MOESM1_ESM.zip › Figure 1/Figure 1A Image data/Hippocampus CA1 - GHF201.jpg]

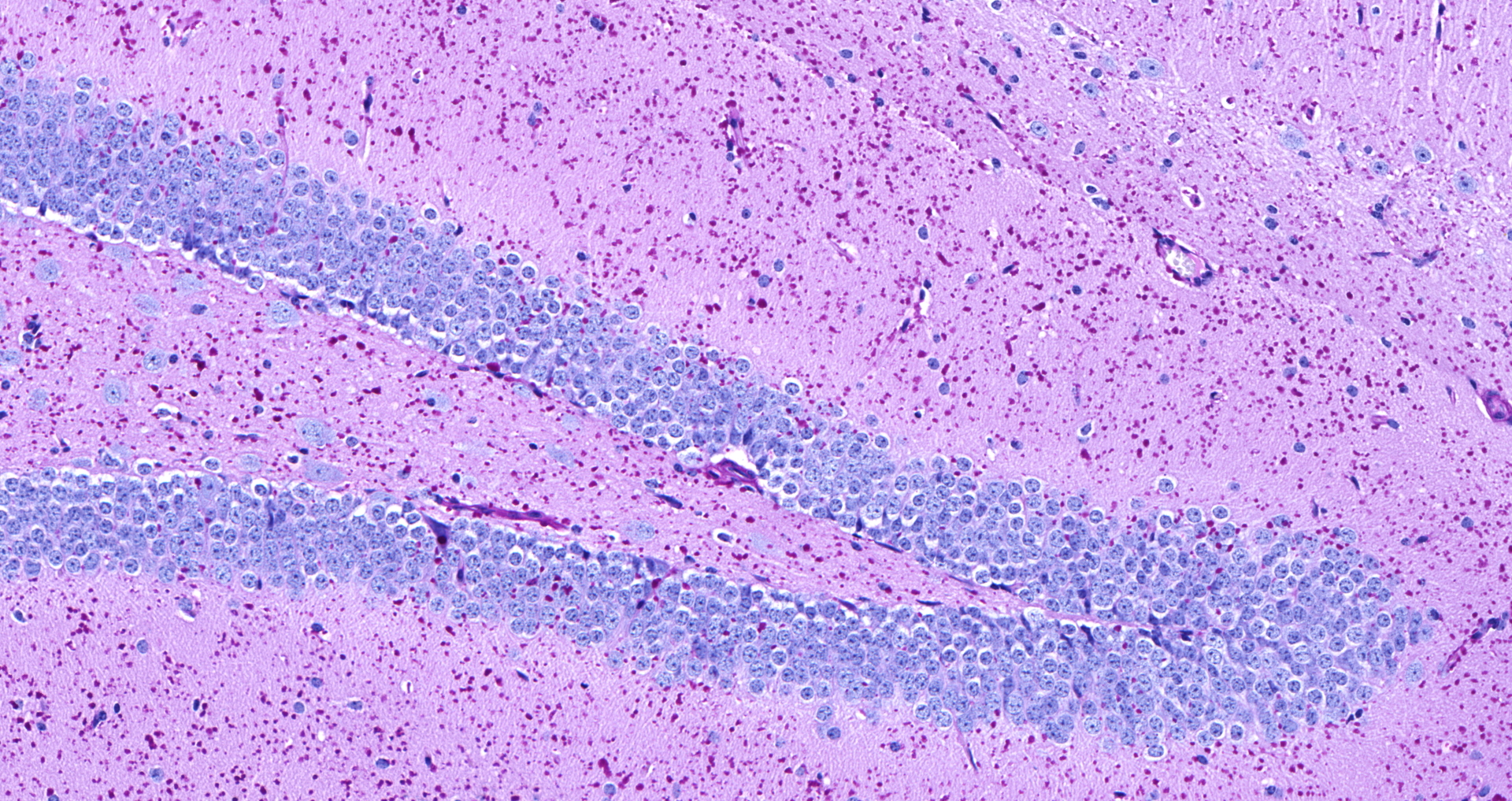

Supplement: Supplementary file 1 — Source data Fig. 1 [file 44321_2024_63_MOESM1_ESM.zip › Figure 1/Figure 1A Image data/Hippocampus DG - Control.jpg]

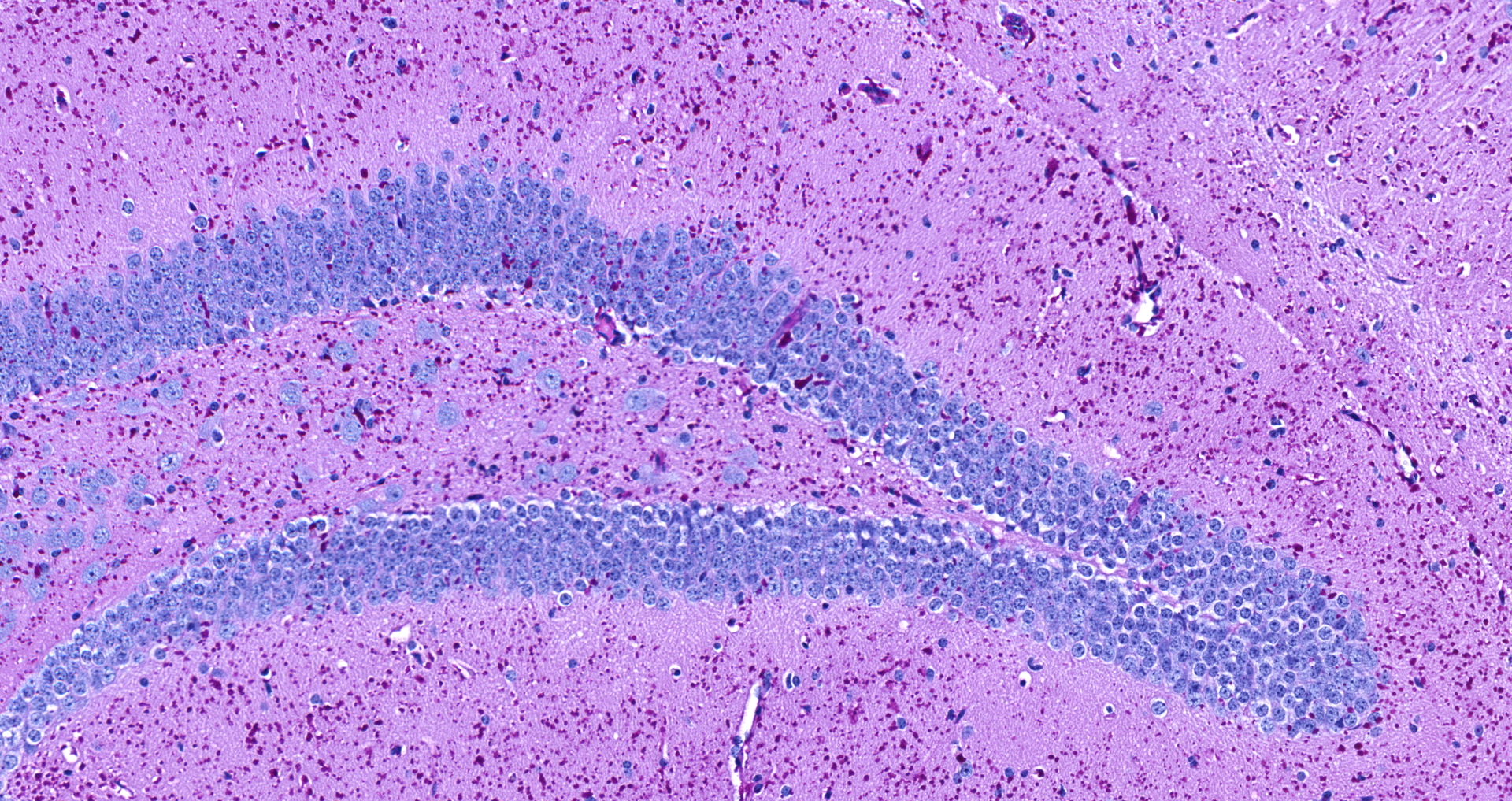

Supplement: Supplementary file 1 — Source data Fig. 1 [file 44321_2024_63_MOESM1_ESM.zip › Figure 1/Figure 1A Image data/Hippocampus DG - GHF201.jpg]

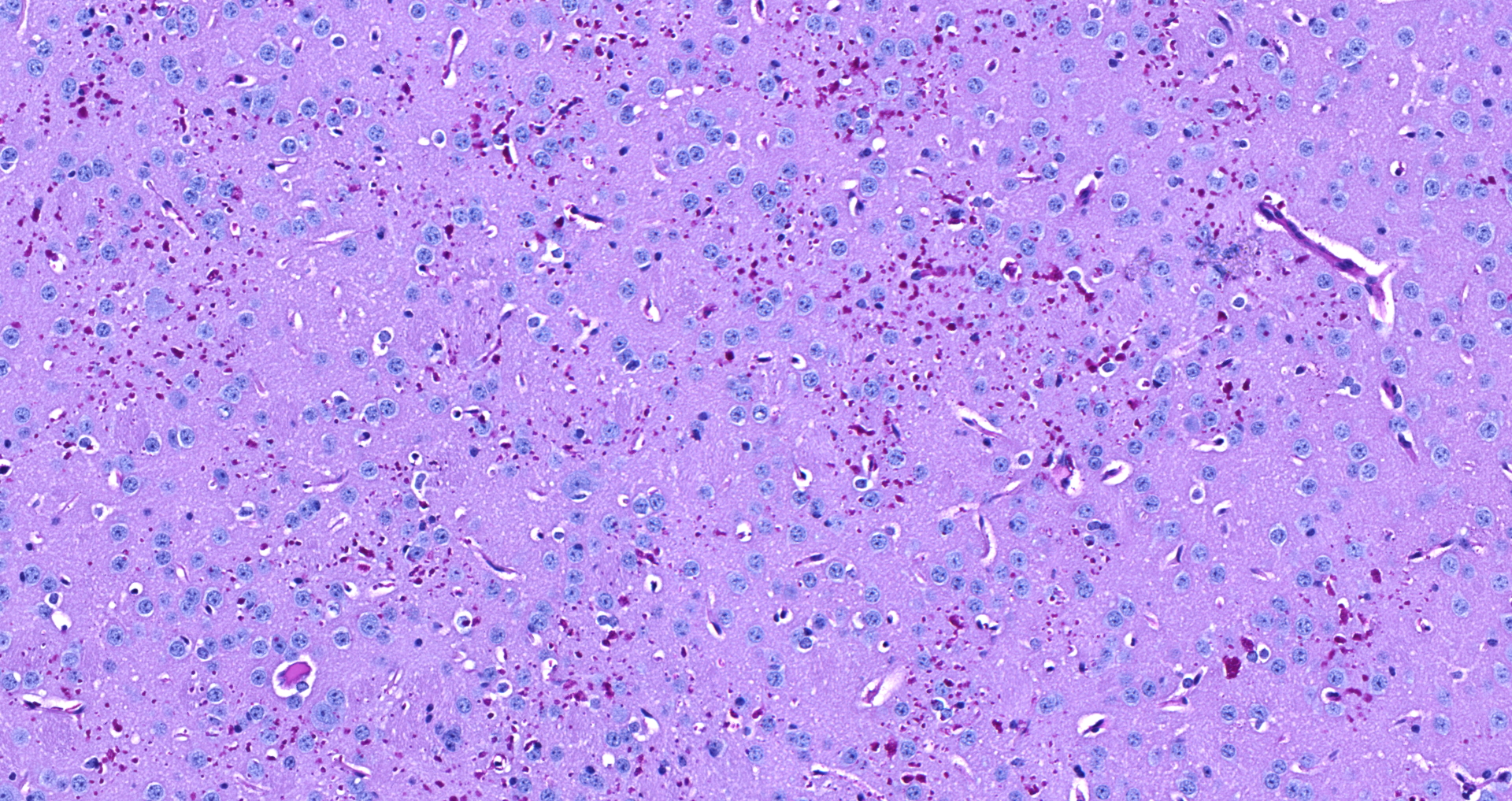

Supplement: Supplementary file 1 — Source data Fig. 1 [file 44321_2024_63_MOESM1_ESM.zip › Figure 1/Figure 1A Image data/Striatum - Control.jpg]

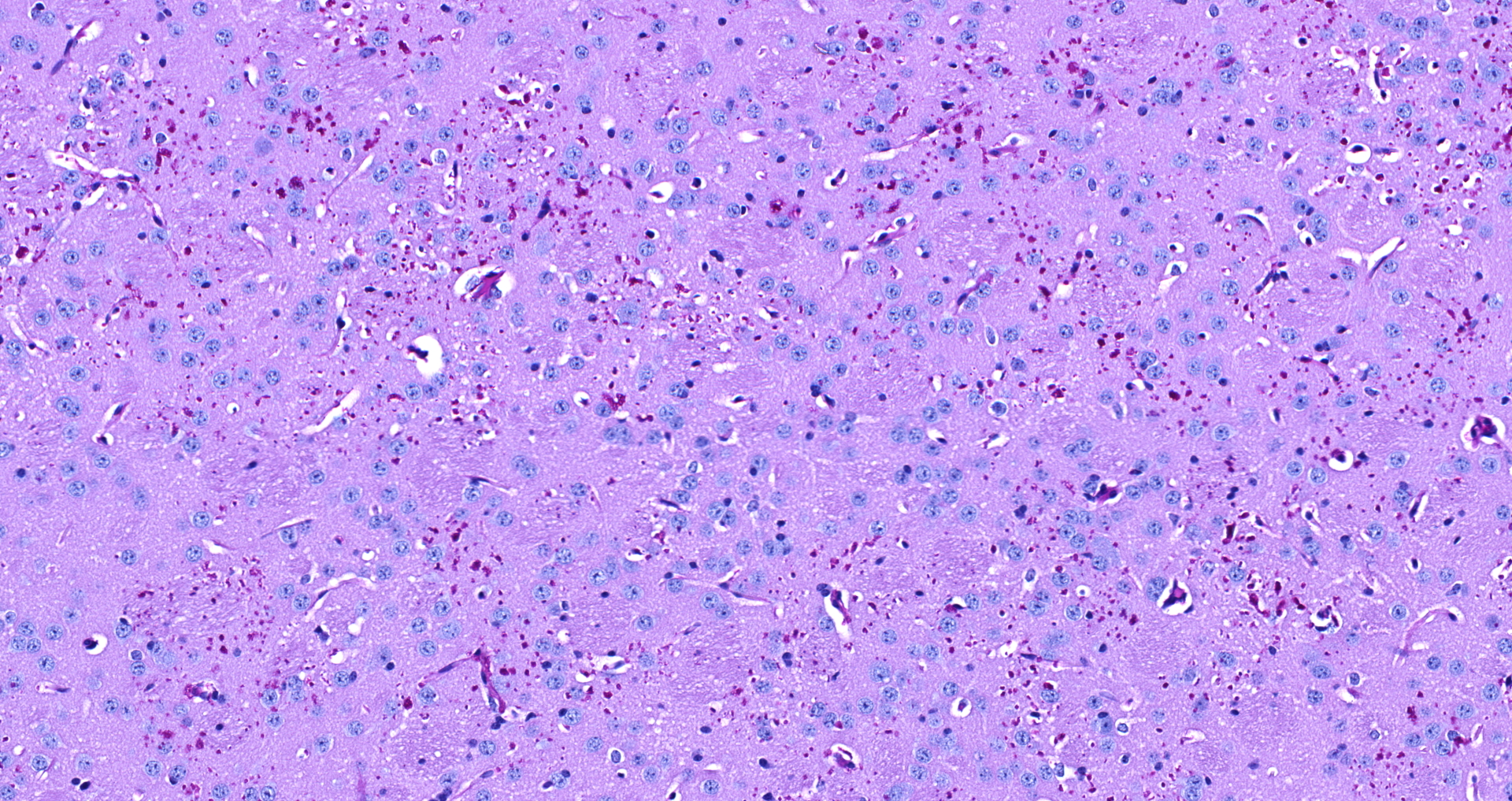

Supplement: Supplementary file 1 — Source data Fig. 1 [file 44321_2024_63_MOESM1_ESM.zip › Figure 1/Figure 1A Image data/Striatum - GHF201.jpg]

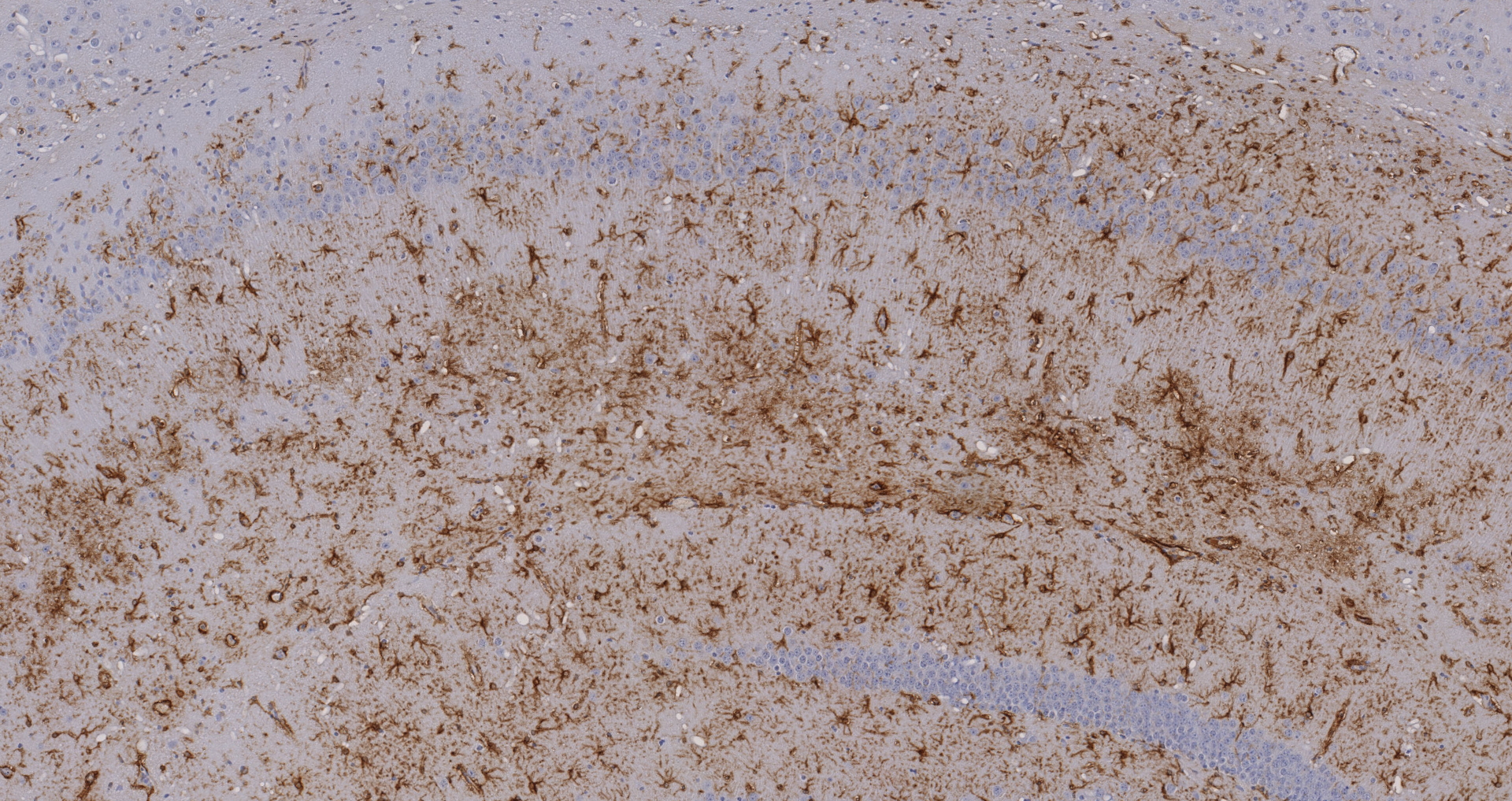

Supplement: Supplementary file 1 — Source data Fig. 1 [file 44321_2024_63_MOESM1_ESM.zip › Figure 1/Figure 1D Image data/Control.jpg]

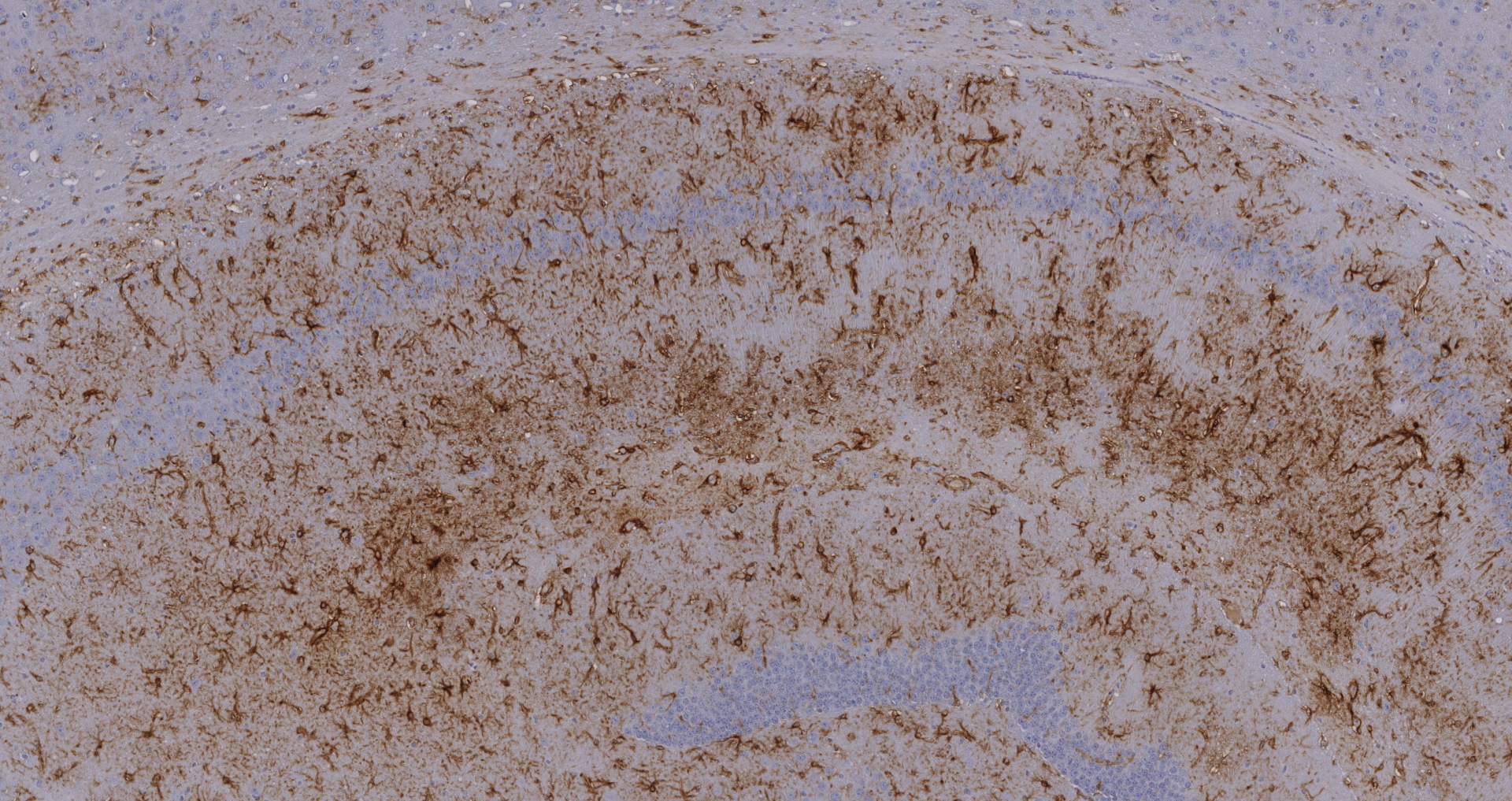

Supplement: Supplementary file 1 — Source data Fig. 1 [file 44321_2024_63_MOESM1_ESM.zip › Figure 1/Figure 1D Image data/GHF201.jpg]

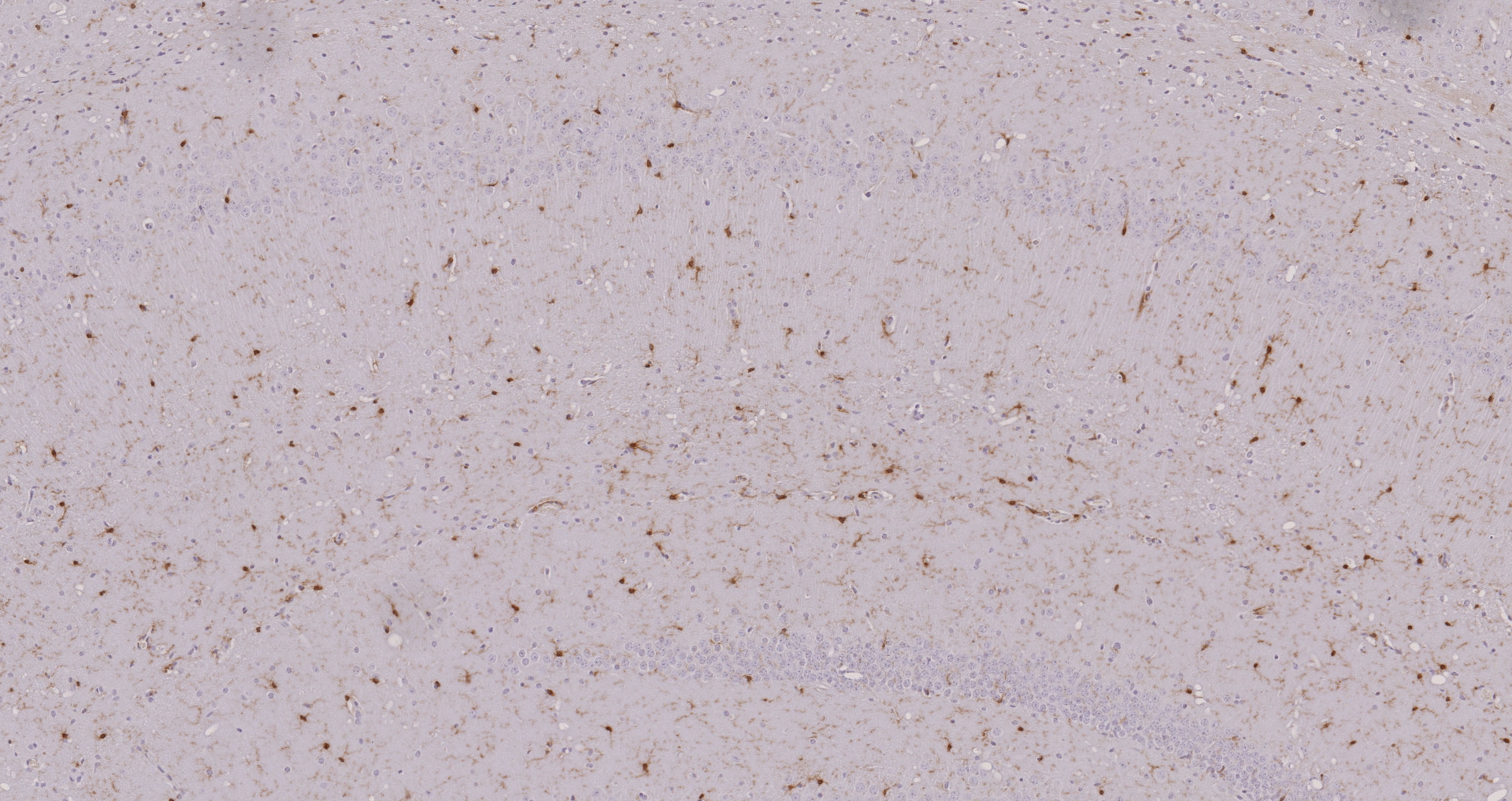

Supplement: Supplementary file 1 — Source data Fig. 1 [file 44321_2024_63_MOESM1_ESM.zip › Figure 1/Figure 1E Image data/Control.jpg]

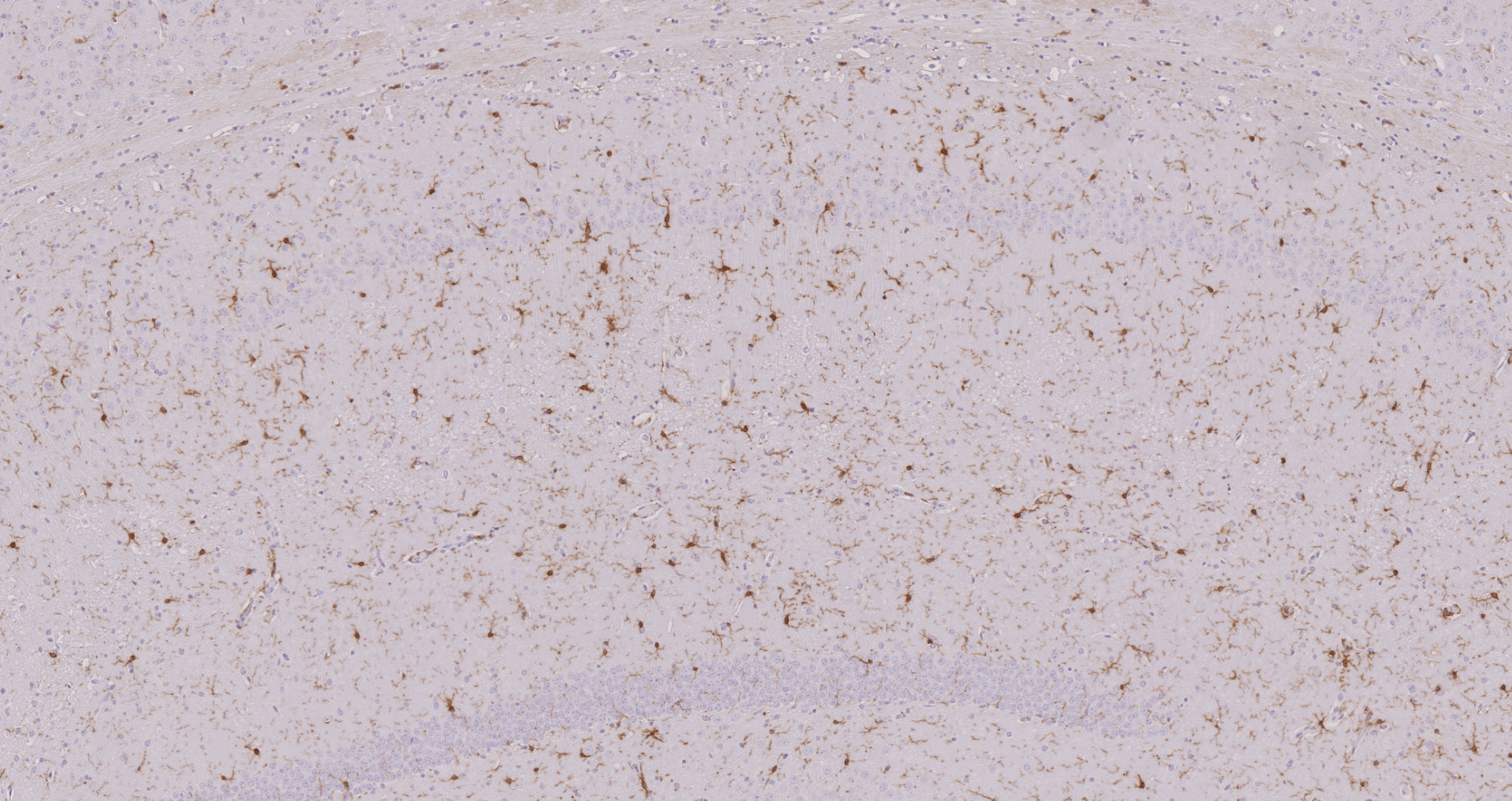

Supplement: Supplementary file 1 — Source data Fig. 1 [file 44321_2024_63_MOESM1_ESM.zip › Figure 1/Figure 1E Image data/GHF201.jpg]
